# Delivery of an Rhs‐family nuclease effector reveals direct penetration of the gram‐positive cell envelope by a type VI secretion system in *Acidovorax citrulli*

**DOI:** 10.1002/mlf2.12007

**Published:** 2022-03-24

**Authors:** Tong‐Tong Pei, Yumin Kan, Zeng‐Hang Wang, Ming‐Xuan Tang, Hao Li, Shuangquan Yan, Yang Cui, Hao‐Yu Zheng, Han Luo, Xiaoye Liang, Tao Dong

**Affiliations:** ^1^ State Key Laboratory of Microbial Metabolism, Joint International Research Laboratory of Metabolic & Developmental Sciences, School of Life Sciences and Biotechnology Shanghai Jiao Tong University Shanghai China; ^2^ Department of Immunology and Microbiology School of Life Sciences, Southern University of Science and Technology Guangdong China

**Keywords:** cell envelope, cell wall, interspecies interaction, protein secretion

## Abstract

The type VI secretion system (T6SS) is a double‐tubular nanomachine widely found in gram‐negative bacteria. Its spear‐like Hcp tube is capable of penetrating a neighboring cell for cytosol‐to‐cytosol protein delivery. However, gram‐positive bacteria have been considered impenetrable to such T6SS action. Here we report that the T6SS of a plant pathogen, *Acidovorax citrulli* (AC), could deliver an Rhs‐family nuclease effector RhsB to kill not only gram‐negative but also gram‐positive bacteria. Using bioinformatic, biochemical, and genetic assays, we systematically identified T6SS‐secreted effectors and determined that RhsB is a crucial antibacterial effector. RhsB contains an N‐terminal PAAR domain, a middle Rhs domain, and an unknown C‐terminal domain. RhsB is subject to self‐cleavage at both its N‐ and C‐terminal domains and its secretion requires the upstream‐encoded chaperone EagT2 and VgrG3. The toxic C‐terminus of RhsB exhibits DNase activities and such toxicity is neutralized by either of the two downstream immunity proteins, RimB1 and RimB2. Deletion of *rhsB* significantly impairs the ability of killing *Bacillus subtilis* while ectopic expression of immunity proteins RimB1 or RimB2 confers protection. We demonstrate that the AC T6SS not only can effectively outcompete *Escherichia coli* and *B. subtilis in planta* but also is highly potent in killing other bacterial and fungal species. Collectively, these findings highlight the greatly expanded capabilities of T6SS in modulating microbiome compositions in complex environments.

## Impact statement

Microbes have developed various weapons to compete against each other for survival. Here, we characterize a type VI secretion system (T6SS) from a plant pathogen that exhibits a greater capability than any other known T6SSs in killing both gram‐negative and gram‐positive bacteria and fungal competitors. The susceptibility of gram‐positive cells to a nuclease effector highlights the power of the T6SS spear to directly penetrate the envelopes of diverse cell types. These findings provide important insights for understanding the broad ecological impact of the T6SS on the dynamic composition and functions of polymicrobial communities.

## INTRODUCTION

To survive in natural and host environments, microbes have developed a number of strategies to outcompete neighboring species, including the production of diffusible molecules, releasing phage or phage‐like particles, and utilization of contact‐dependent antibacterial weapons^1^, ^2^, ^3^, ^4^, ^5^, ^6^, ^7^, ^8^. One such lethal weapon is the type VI secretion system (T6SS) that gram‐negative bacteria commonly employ to deliver toxins into neighboring cells through a phage‐tail‐like double tubular contractile structure^7^, ^9^, ^10^. The T6SS ejects a spear‐like tube with toxic effectors outward in milliseconds, with enough power to drill through the cell wall and two cellular membranes of gram‐negative cells^10^, ^11^, ^12^. T6SSs show great diversity in the arsenal of toxic effectors and, as a result, potent killing capabilities against susceptible bacteria, amoeba, yeast, and other eukaryotic cells^7^, ^13^, ^14^, ^15^, ^16^, ^17^.

To defend against the T6SS attack, bacteria have developed both specific and nonspecific protective mechanisms^15^, ^18^, ^19^. The effector‐specific protection is mediated by effector‐cognate immunity proteins that can neutralize effectors through interaction^15^, ^20^, ^21^. However, such specific protection can be insufficient in the absence of bacterial innate‐immunity‐like nonspecific mechanisms^18^, ^19^, including the production of exopolysaccharide (EPS) capsules, oxidative stress response, and envelope stress responses^19^, ^22^, ^23^, ^24^. Notably, most T6SS‐susceptible bacteria are gram‐negative, while gram‐positive bacteria are not generally believed to be targets of T6SS^25^, ^26^, ^27^. The T6SS resistance of gram‐positive cells has been largely attributed to a stronger envelope consisting of thicker peptidoglycan layers, polymers of teichoic acids, and surface proteins, which may present an impenetrable barrier to the T6SS spear^28^. Although T6SS‐secreted cell‐wall‐lysing effectors have been shown effective against gram‐positive *Bacillus subtilis* cells^26^, ^29^, and the T6SS of *Pseudomonas chlororaphis* appears to induce spore formation of *B. subtilis* ^30^, it remains elusive whether the T6SS is capable of penetrating into gram‐positive cells to achieve direct cytosol‐to‐cytosol delivery.

*Acidovorax citrulli* (AC) is a gram‐negative seed‐borne plant pathogen that causes bacterial fruit blotch of cucurbit crops (including melons and watermelons) and is extremely difficult to eradicate^31^. Similar to many human pathogens, *A. citrulli* has rapidly spread globally in the last few decades and is the most economically important pathogen to cucurbits^31^, ^32^. *A. citrulli* possesses a number of virulence traits, of which the type III secretion system is required for infection^33^ and the T6SS appears to contribute to seed‐to‐seedling transmission^34^. Here, we report that the T6SS of *A. citrulli* type strain AAC00‐1 possesses potent antibacterial activities against gram‐negative and gram‐positive bacteria. Using secretome, bioinformatic, and genetic‐screening analyses, we characterized T6SS‐dependent effectors in AC and determined two Rhs (rearrangement hot spot) family effectors, RhsB and RhsE, that are crucial for outcompeting *B. subtilis*. We report that RhsB is a nuclease effector whose secretion is mediated by the upstream‐encoded chaperone EagT2 and VgrG3. We reveal that RhsB is subject to self‐cleavage that is crucial for its toxicity. In summary, our results demonstrate that the T6SS of AC could breach the thick cell envelope of gram‐positive *B. subtilis* and deliver the RhsB nuclease to *B. subtilis* in a cytosol‐to‐cytosol manner. The diverse antibacterial and antifungal T6SS functions in the plant pathogen *A. citrulli* highlight an expanded ecological impact of the T6SS in multispecies communities far beyond the pathogenesis of human diseases.

## RESULTS

### AC T6SS displays potent antibacterial and antifungal activities

**Figure 1:**
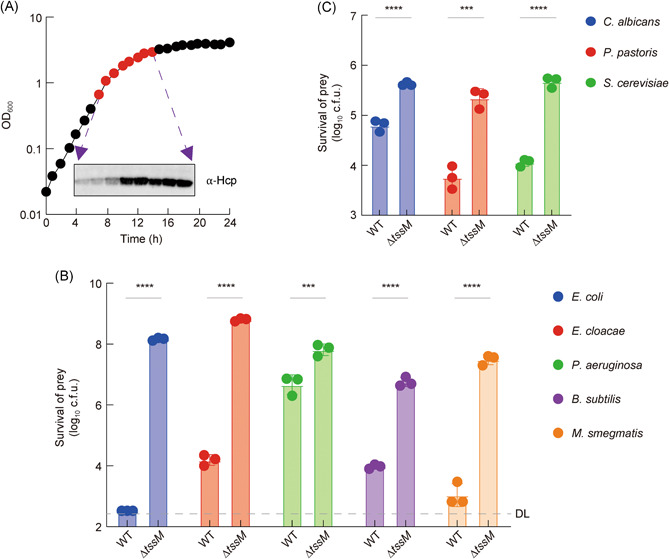
The T6SS of *A. citrulli* displays antibacterial and antifungal activities. (A) Growth curve of AC at 28°C. The secretion levels of Hcp were examined during growth. Competition assay of wild type (WT) and the T6SS‐null Δ*tssM* mutant against a panel of bacterial (B) and fungal (C) strains. Survival of prey cells was determined by serial dilutions on selective media. Error bars indicate standard deviation of three biological replicates and statistical significance was calculated using a two‐tailed Student's *t*‐test for each group, ***p < 0.001, ****p < 0.0001. DL, detection limit.

To test whether the *A. citrulli* AAC00‐1 strain (AC) possesses a functional T6SS, we first examined the expression and secretion of Hcp that forms the shaft of the T6SS spear‐like secreted structure^9^, ^10^. We found that Hcp was well expressed during aerobic growth in LB but its secretion was increased substantially during the transition from exponential to stationary phase growth (Figures 1A and S1A). These results indicate that the T6SS of AC is active and functional under lab conditions.

Next, we tested whether the T6SS of AC exhibits antibacterial and antifungal activities as previously reported for other T6SS systems^13^, ^15^, ^25^. First, we constructed a T6SS‐null mutant lacking the *tssM* gene that encodes an essential T6SS inner membrane protein^7^. Using interspecies competition assays, we compared the survival of different prey species against wild type or the ∆*tssM* mutant of AC. Results show that the T6SS could effectively kill bacterial competitors *Escherichia coli*, *Enterobacter cloacae, P. aeruginosa, B. subtilis*, and *Mycobacterium smegmatis*, as well as fungal competitors *Pichia pastoris*, *Saccharomyces cerevisiae*, and *Candida albicans* (Figures 1B,C and S1,B–I). These prey strains exhibited variable susceptibilities to the T6SS ranging from several‐fold reduction for *C. albicans* to near one‐million‐fold reduction in survival for *E. coli* between wild‐type and the ∆*tssM* samples (Figure 1B,C). Collectively, the T6SS of AC targets a wide range of microbial competitors.

### T6SS deploys multiple effectors

**Figure 2:**
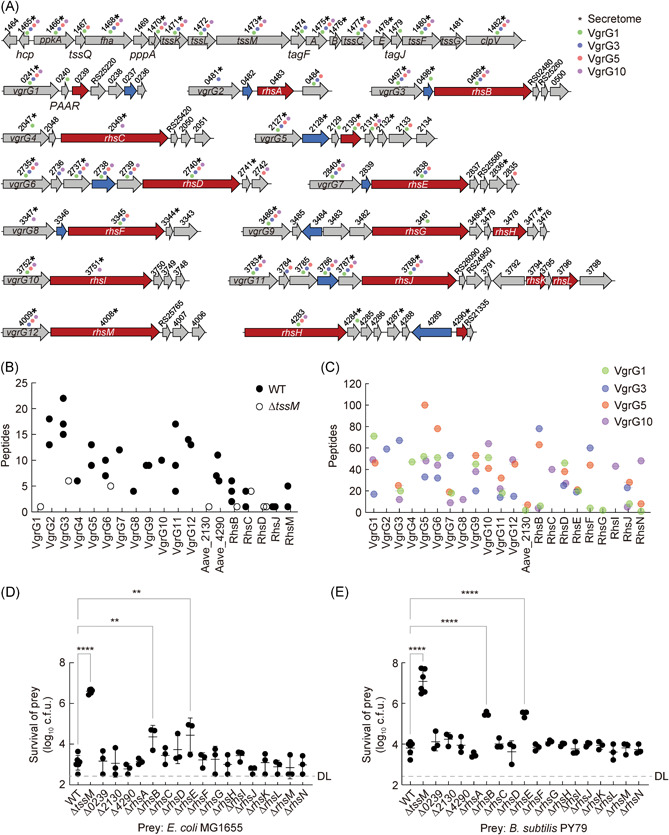
Identification of T6SS effectors. (A) T6SS gene cluster and operons. Predicted effector and chaperone genes are shown in red and blue, respectively. Asterisks indicate the corresponding proteins detected in the secretome results of (B) and Table S1. Different colors of dots indicate the corresponding proteins pulled down with VgrG1, VgrG3, VgrG5, and VgrG10 shown in (C) and Table S2. (B) Secretome analysis summarizing the detected VgrG and effector proteins between the WT and the T6SS‐null mutant (Δ*tssM*). (C) Pull‐down analysis summarizing VgrG and effector proteins pulled down with VgrG1, VgrG3, VgrG5, and VgrG10. For (B) and (C), all represented proteins were detected with unique peptides. The Y‐axis shows the number of peptides detected of the corresponding protein shown in X‐axis, while the specific number of peptides, unique peptides, and coverage are shown in Tables S1 and S2, respectively. Survival of *E. coli* MG1655 (D) and *B. subtilis* PY79 (E) attacked by AAC00‐1 WT, Δ*tssM*, and effector inactivation mutants for 1 and 3 h, respectively. Survival of prey cells was determined by serial dilutions on selective media. Error bars indicate the standard deviation of at least three biological replicates and statistical significance was calculated using one‐way ANOVA test for each group, **p < 0.01, ****p < 0.0001. DL, detection limit.

Because effectors dictate T6SS functions, we next set to systematically examine T6SS effectors in *A. citrulli*. Genome analysis reveals that AC contains a large T6SS cluster encoding mostly structural components and 13 smaller operons, encoding T6SS‐secreted spike VgrG/PAAR proteins or conserved Rhs‐family proteins^35^, ^36^, ^37^, ^38^ (Figure 2A). Using the known genetic linkage of VgrG/PAAR to downstream effectors and the Rhs conserved signature^16^, ^36^, ^37^, ^39^, we predicted 17 effector genes, 14 of which belong to the Rhs family (Figure 2A).

To validate the predicted effectors, we first employed a secretome approach using LC‐MS/MS analysis to compare the secreted proteins between wild type and the ∆*tssM* mutant. We successfully detected unique peptides corresponding to Hcp, all the 12 VgrG proteins, and 7 of the predicted T6SS effectors (Figure 2B and Table S1). These results suggest that the small T6SS operons are also actively expressed, similar to the large cluster.

Because VgrG proteins often directly interact with their cognate effectors for secretion, we also used pull‐down assays with a select group of VgrG proteins as bait to determine their interacting effectors. We expressed plasmid‐borne N‐terminal 6His‐tagged VgrG1, VgrG3, VgrG5, and VgrG10, respectively, in AC and analyzed the eluted proteins from His‐affinity columns using LC‐MS/MS analysis. Using this approach, we detected nine Rhs‐family effectors, suggesting it is an effective approach to identify VgrG‐dependent effectors (Figure 2C and Table S2).

### RhsB and RhsE are critical for T6SS antibacterial activities

To determine whether these effectors contribute to interspecies competition, we constructed a panel of effector mutants by deletion or insertion. Using bacterial competition assays against *E. coli* and *B. subtilis* preys, we screened these effector mutants and found that, while most mutants exhibited wild‐type level killing abilities against prey cells, two mutants (Aave_0499, named RhsB and Aave_2838, named RhsE) showed impaired killing activities (Figures 2D,E and S2A,B). To test whether deletion of *rhsB* or *rhsE* affects T6SS secretion, we compared Hcp secretion of these two mutants with the wild type. Results show that mutants of *rhsB* and *rhsE* had wild‐type level of Hcp secretion (Figure S2C). Collectively, these results suggest RhsB and RhsE are key antibacterial effectors.

### RhsB is neutralized by its downstream immunity proteins RimB1&2

**Figure 3:**
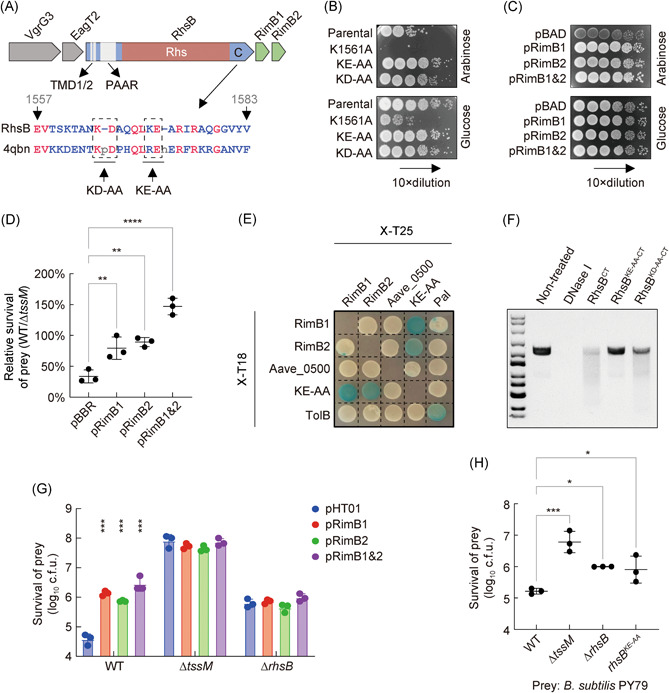
Characterization of RhsB function. (A) Operon structure of *rhsB*. The sequence of the RhsB C‐terminus is aligned with the sequence of a virus‐type replication‐repair nuclease domain (VRR‐Nuc, PDB: 4qbn). (B) Toxicity of expressing RhsB C‐terminus (Parental) and its mutants in *E. coli*. (C) Toxicity of expressing RhsB with an empty vector (pBAD) or a vector carrying the immunity gene *rimB1*, *rimB2*, or *rimB1* and *rimB2* together as indicated in *E. coli*. For (B) and (C), all constructs were cloned on pBAD vectors and survival of *E. coli* was enumerated by serial plating on arabinose (induction) and glucose (repression) plates. (D) Competition assay of WT and the T6SS‐null Δ*tssM* mutant against the effector–immunity deletion mutant Δ*rhsB*‐*rimB1&2* complemented with an empty vector (pBBR) or a vector expressing the immunity protein RimB1, RimB2, or RimB1 and RimB2 together as indicated. The data point indicates the relative survival of prey cells attacked by WT compared with that by T6SS mutant. (E) Bacterial two‐hybrid analysis of RhsB–RimB1&2 interaction. Fusion proteins with T18 and T25 domains were co‐expressed in the reporter strain BTH101 as indicated. (F) DNA degradation by RhsB C‐terminus (RhsB^CT^) and its mutants. The qualities of purified proteins are shown by SDS‐PAGE analysis in Figure S3C. (G) Competition assay of WT, Δ*tssM* and Δ*rhsB* mutant against the *B. subtilis* PY79 carrying an empty vector (pHT01) or a vector expressing the immunity protein RimB1, RimB2 or RimB1 and RimB2 together as indicated. Cells of killer and prey were mixed at a ratio of 20:1 (killer:prey) and spotted on LB agar plates with 1 mM IPTG for 3 h at 37°C. (H) Competition assay of WT, Δ*tssM*, Δ*rhsB*, and *rhsB* ^*KE‐AA*^ mutant against the *B. subtilis* PY79. For (D), (G), and (H), error bars indicate the standard deviation of three biological replicates and statistical significance was calculated using a one‐way ANOVA test, *p < 0.05, **p < 0.01, ***p < 0.001, ****p < 0.0001.

Both *rhsB* and *rhsE* encode Rhs‐family proteins with an N‐terminal PAAR domain, a middle YD‐repeat/Rhs domain, and a C‐terminal domain of unknown function (Figure 3A). Using Phyre2 sequence analysis, we found no significant hit for RhsE but the C‐terminal domain of RhsB is distantly related to a virus‐type replication‐repair nuclease (PDB: 4qbn) with 24% identity^40^. Downstream of *rhsB* reside two small predicted genes of unknown functions sharing 69% identity and equal length in the protein sequence. We name the two downstream genes *rimB1* and *rimB2* (Rhs‐immunity B), respectively.

To test if the C‐terminus of RhsB (RhsB^CT^) is toxic, we expressed it using an arabinose inducible vector (pBAD) in *E. coli* and compared cell survival in the presence of arabinose (induced) or glucose (repressed)^41^. Results show that RhsB^CT^ was highly toxic, reducing survival by 100 folds when induced (Figure 3B). Although RhsB^CT^ belongs to the PD‐(D/E)XK superfamily^42^, there are no obvious catalytic sites in 4qbn or RhsB^CT^. We constructed several point mutations around the PD site and tested their toxicity in *E. coli*. Both the KE‐AA mutant and the KD‐AA mutant displayed severely attenuated toxicity compared with the wild‐type RhsB^CT^ (Figure 3B). Interestingly, the K1561A mutant exhibited stronger toxicity than its parental protein (Figure 3B). And the results of Western blot analysis confirmed that these mutant proteins were expressed (Figure S3A).

To test whether RimB proteins confer protection, we first expressed RhsB with and without the downstream genes in *E. coli* using the arabinose‐inducible pBAD vector. Results show that RhsB toxicity can be neutralized by co‐expression of RimB1 or RimB2 similarly (Figure 3C). We also constructed the ∆*rhsB‐rimB1&2* mutant lacking the immunity genes and transformed it with an empty vector or vectors encoding one or both immunity proteins. Competition analyses against wild‐type AC or the ∆*tssM* mutant reveal that survival was significantly increased when immunity genes were ectopically expressed in the ∆*rhsB‐rimB1&2* mutant (Figures 3D and S3B). Using bacterial two‐hybrid assays^16^, ^43^, we found that both RimB1 and RimB2 could bind to the nontoxic RhsB^KE‐AA^ construct, while the Aave_0500 protein, encoded downstream of *rimB2*, did not interact with any of the proteins (Figure 3E). As control, Pal and TolB proteins showed positive interaction^44^, ^45^. These results collectively suggest both immunity proteins could confer protection. To examine if RhsB exhibits nuclease activity, we purified the C‐terminal domain of RhsB and its inactive mutants RhsB^KE‐AA^ and RhsB^KD‐AA^, all with an N‐terminal 6His‐SUMO tag. Results show that only the wild‐type RhsB^CT^ but not the two catalytically inactive mutants could efficiently degrade DNA (Figures 3F and S3C).

Collectively, these results indicate that RhsB is a nuclease effector and has two cognate immunity proteins RimB1 and RimB2.

### RhsB is delivered into the cytosol of *B. subtilis*

Next, we tested whether RhsB is delivered to *B. subtilis* using a competition assay. The prey *B. subtilis* was transformed with the empty vector pHT01 or vectors expressing one or both immunity proteins, separately. Results show that all *B. subtilis* strains expressing the immunity genes survived significantly better than the one expressing the vector alone when they competed with wild‐type AC (Figures 3G and S3D). When the ∆*rhsB* mutant was used as the killer, survival of *B. subtilis* with vector alone was increased to a similar level to that of *B. subtilis* expressing immunity genes. In addition, to test whether RhsB could be translocated into *B. subtilis* through diffusion, we co‐incubated *B. subtilis* with wild‐type AC or the ∆*tssM* mutant in liquid culture, respectively. There was no significant difference in *B. subtilis* survival (Figure S3E,F). These results collectively indicate that RhsB is delivered by the T6SS to *B. subtilis* and its toxicity is neutralized by the RimB immunity proteins expressed in *B. subtilis*. We also constructed the chromosomal nuclease‐inactivated mutant RhsB^KE‐AA^ and found this mutant also exhibited attenuated killing ability of *B. subtilis* to the same level as deletion of *rhsB* did (Figure 3H), suggesting RhsB intoxicated *B. subtilis* due to its nuclease activity.

### RhsB secretion is mediated by the chaperone EagT2 and VgrG3

**Figure 4:**
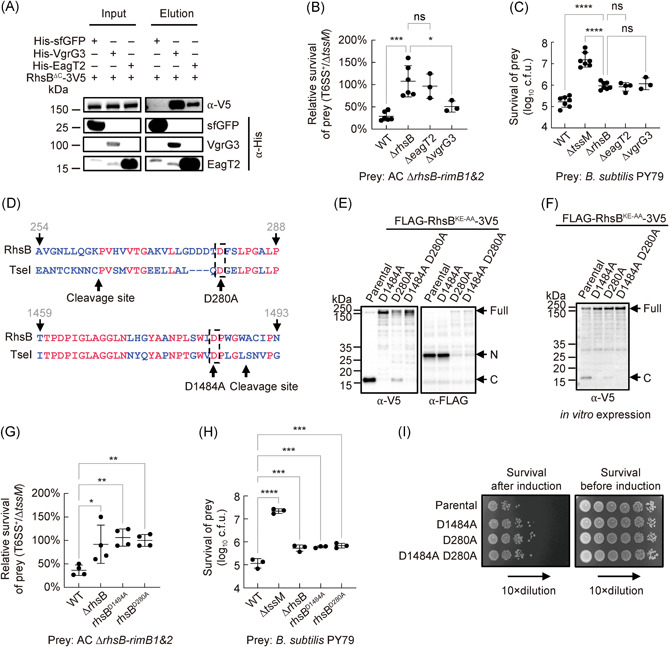
Secretion and cleavage of RhsB. (A) Interaction of RhsB^∆C^ with VgrG3 and chaperone EagT2. Pull‐down analysis was performed using His‐tagged sfGFP (control), VgrG3 or EagT2 and V5‐tagged RhsB^∆C^. Competition assay of WT, Δ*tssM*, Δ*rhsB*, Δ*eagT2*, and Δ*vgrG3* mutants against the Δ*rhsB*‐*rimB1&2* (B) and *B. subtilis* PY79 (C). (D) Alignment of the predicted sequences of N‐terminus and C‐terminus cleavage regions between RhsB and TseI. (E) Western blot analysis of RhsB^KE‐AA^ and its cleavage‐defective mutants D1484A, D280A, and D1484A D280A. Proteins were induced in *E. coli* with IPTG (0.01 mM). The nontoxic KE‐AA mutant is used as the parental construct. The same constructs were also used for *in vitro* expression shown in (F). (F) *In vitro* expression of RhsB^KE‐AA^ and its cleavage‐defective mutants D1484A, D280A, and D1484A D280A. The proteins were expressed using a PURExpress^®^ In Vitro Protein Synthesis Kit. Samples were subject to SDS‐PAGE analysis, followed by Western blot analysis. Competition assay of WT, Δ*tssM*, Δ*rhsB*, *rhsB* ^*D1484A*^, and *rhsB* ^*D280A*^ mutant against the Δ*rhsB*‐*rimB1&2* (G) and *B. subtilis* PY79 (H). (I) Toxicity of expressing RhsB and its cleavage‐defective mutants in *E. coli*. All constructs were cloned on pBAD vectors and the survival of *E. coli* after arabinose induction was examined by serial plating on plates. For the relative survival in (B) and (G), it was calculated by using the final survival of the prey cells competed with different T6SS‐active killer strains (T6SS^+^) divided by the survival of prey cells competed with the ∆*tssM* mutant. For (B), (C), (G), and (H), error bars indicate the standard deviation of at least three biological replicates and statistical significance was calculated using a one‐way ANOVA test for each group, *p < 0.05, **p < 0.01, ***p < 0.001, ****p < 0.0001, ns, not significant.

RhsB is encoded in a gene cluster with its immediate upstream genes encoding VgrG3 and a DUF1795‐domain chaperone EagT2^46^, ^47^. To determine whether these two proteins are important for RhsB secretion, we first performed a pull‐down analysis using the nontoxic RhsB^ΔC^ mutant that lacks the C‐terminus toxin domain. The results confirmed that RhsB could directly interact with EagT2 and VgrG3 (Figures 4A and S4A). We next constructed the deletion mutants of *eagT2* and *vgrG3* and tested their killing abilities against the ∆*rhsB‐rimB1&2* prey cells. The ∆*eagT2* displayed a killing defect similar to the ∆*rhsB* mutant, suggesting that EagT2 is crucial for RhsB secretion (Figures 4B and S4B). In contrast, the ∆*vgrG3* mutant showed impaired killing ability relative to wild type but still significantly stronger killing ability than the ∆*rhsB* mutant (Figures 4B and S4B). Considering the ∆*rhsB‐rimB1&2* cells possess active T6SS and could donate VgrG3 but not EagT2 into sister cells, we postulated that the partial defect in the ∆*vgrG3* mutant may result from substrate exchange between T6SS neighboring cells, a known phenotype previously described in *Vibrio cholerae* ^11^. Indeed, when we performed competition analysis against the *B. subtilis* prey, results show that the ∆*eagT2* and the ∆*vgrG3* mutants exhibited impaired killing activities similar to the ∆*rhsB* mutant (Figures 4C and S4C).

### RhsB is self‐cleaved at both N‐ and C‐terminus

We have previously described a self‐cleavable Rhs effector TseI in *Aeromonas dhakensis* ^36^. Although RhsB possesses an N‐terminal PAAR domain that is absent in TseI, sequence alignment of these two proteins shows that RhsB contains the conserved aspartic cleavage residues around the putative cleavage sites for both N‐ and C‐terminal sequences (Figure 4D and Supporting Information Data 1).

To test whether RhsB is cleaved, we expressed the nontoxic RhsB^KE‐AA^ mutant protein in *E. coli* since the wild type is highly toxic. Western blot analysis shows that the N‐terminus and the C‐terminus of RhsB^KE‐AA^ were also cleaved (Figure 4E). Mutation of the conserved residue D1484 to alanine abolished C‐terminal cleavage while the D280 to alanine mutation impaired both N‐ and C‐terminal cleavage (Figure 4E). We also performed *in vitro* expression of RhsB using the In Vitro Protein Synthesis kit consisting of defined transcription‐translation protein components. Western blot analysis of *in vitro* expressed RhsB confirms that RhsB is self‐cleaved at the C‐terminus while the N‐terminal cleavage is indiscernible due to high background noise signals under the *in vitro* expression condition (Figures 4F and S4D). In addition, we detected RhsB cleavage in the cytosol of *A. citrulli* by expressing its nontoxic mutant RhsB^KE‐AA^ in AC wild type, T6SS‐null mutant ∆*tssM*, and ∆*rhsB*, suggesting that self‐cleavage of RhsB could occur before secretion and be independent of T6SS functionality (Figure S4E).

In addition, we purified the His‐SUMO‐tagged RhsB^KE‐AA^ with His‐tagged EagT2 together, while expressing RhsB^KE‐AA^ alone had much less yield. The purified N‐terminal His‐SUMO‐tagged RhsB^KE‐AA^ protein exhibited clear cleavage products (Figure S4F). Using N‐terminal protein sequencing, we determined the C‐terminal cleavage site after the residue tryptophan 1488, which is consistent with the predicted site based on the sequence alignment (Figure 4D). However, the N‐terminal cleavage site was unsolved due to poor detection by N‐terminal sequencing. Nonetheless, Western blot analysis of the predicted N‐terminus (RhsB^NT^) expressed in *E. coli* shows a nearly‐identical size to the cleaved N‐terminal product from full‐length RhsB (Figure S4G). The predicted cleavage site of RhsB is also consistent with the recently reported N‐terminal cleavage site of Rhs1 in *Photorhabdus laumondii* ^48^.

### Cleavage is crucial for RhsB‐mediated toxicity

To test whether cleavage is important for RhsB functions, we first examined RhsB‐dependent intra‐ and inter‐species competition. Results show that the cleavage‐defective mutants *rhsB* ^*D280A*^ and *rhsB* ^*D1484A*^ displayed impaired killing abilities, similar to the ∆*rhsB* mutant, against the ∆*rhsB‐rimB1&2* and *B. subtilis* prey strains (Figures 4G,H and S4H,I). To test whether cleavage also affects the intracellular toxicity of RhsB, we expressed the full‐length RhsB and its cleavage‐defective mutants using arabinose‐inducible pBAD vectors in *E. coli* and compared their effects on survival after arabinose induction. The results show that the survival of cells expressing wild‐type RhsB was reduced by 10 folds compared with that of the cells expressing the cleavage‐defective mutants (Figure 4I). Collectively, these data show that cleavage is crucial for RhsB‐mediated intracellular toxicity and competition.

### AC employs its T6SS to outcompete other bacteria *in planta*

**Figure 5:**
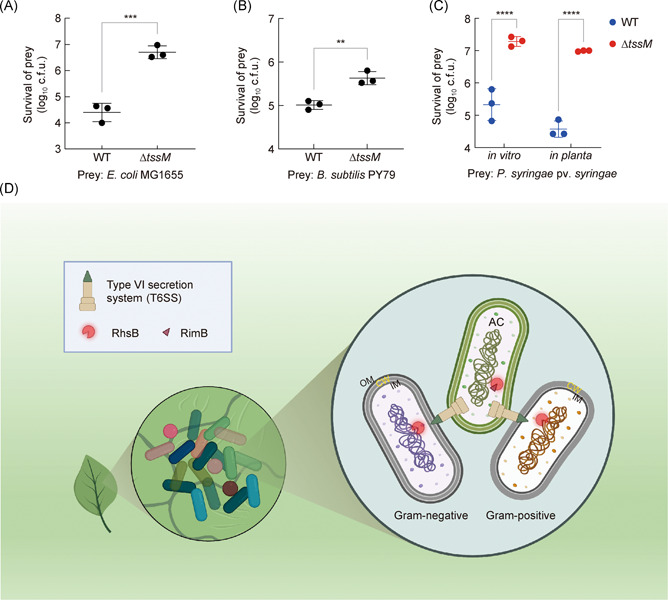
T6SS‐mediated competition *in planta*. Competition assay of WT and Δ*tssM* against the *E. coli* MG1655 (A) and *B. subtilis* PY79 (B). (C) Competition assay of WT and Δ*tssM* against *P. syringae* pv. *syringae* *in vitro* and *in planta*, respectively. Error bars indicate the standard deviation of three biological replicates and statistical significance was calculated using a two‐tailed Student's *t*‐test for each group, **p < 0.01, ***p < 0.001, ****p < 0.0001. (D) Schematic of the T6SS‐dependent killing of AC against gram‐negative and gram‐positive competitors *in planta*. CW, cell wall; IM, inner membrane; OM, outer membrane.

To explore whether the T6SS confers competitive fitness *in planta*, we performed competition assays between AC and *E. coli* MG1655 or *B. subtilis* PY79 using the *Nicotiana benthamiana* leaf infection model^49^. The results show that wild‐type AC could effectively outcompete prey cells in comparison with the ∆*tssM* mutant (Figures 5A,B and S5A,B). Because *E. coli* and *B. subtilis* strains are not natural plant pathogens, we next tested whether the T6SS of AC can kill *P. syringae* pv. *syringae*, which is one of the most common plant pathogens that infect the phyllosphere^50^. Through performing competition assay *in vitro* and *in planta* (Figures 5C and S5C), we found that AC significantly inhibited the growth of *P. syringae* in both conditions. We also validated the T6SS‐mediated competition between AC and *E. coli* using a natural host of AC infection, *Citrullus lanatus* (Figure S5D,E). Collectively, these results indicate that the T6SS of AC is functional and promotes its fitness during plant infection.

## DISCUSSION

In conclusion, we report the potent activities of *A. citrulli* T6SS against a panel of bacterial and fungal species. We have systematically identified the T6SS effectors in *A. citrulli* and determined two Rhs‐family effectors, RhsB and RhsE, which contribute the most to killing both gram‐negative and gram‐positive bacteria. We have determined key residues and interacting proteins that are required for the activity, cleavage, and secretion of RhsB. We further show that the AC T6SS is active against competing species *in planta*. These data underscore the broad ecological impact of the T6SS on diverse natural microbial communities, beyond the previously known susceptible microbes and pathogens of human infections (Figure 5D).

These findings have several important implications. First, in comparison with previous efforts focused on human pathogens, T6SS functions are substantially less studied in plant pathogens despite the vast number of effectors, distinct ecological environments, and enormous impact on world economy and food security. Previous studies have reported key antibacterial functions of the T6SS in several plant‐associated bacteria, including *Agrobacterium tumefaciens*, *Burkholderia glumae*, and *P. protegens* ^51^, ^52^, ^53^, but all are limited to inhibiting gram‐negative bacteria. Our findings will likely stimulate further research in novel T6SS functions of plant pathogens.

Second, our findings address a long‐standing critical question on whether contraction of the T6SS sheath is powerful enough to drill through the thick gram‐positive cell envelope^10^, ^28^. A recent report shows that the T6SS of *Acinetobacter baumannii* could secrete a cell‐wall‐lysing effector Tse4 to kill gram‐positive bacteria^29^. However, it remains unclear whether the secreted Tse4 destroys the gram‐positive cell wall from the outside or from inside, or whether Tse4 can be delivered into the cytosol of gram‐positive cells. Since expressing immunity proteins in *B. subtilis* confers protection against the delivered RhsB nuclease, our study provides the first evidence that the T6SS of AC could penetrate through the cell envelope and achieve cytosol‐to‐cytosol delivery into gram‐positive cells. Notably, other effectors provide redundant functions to RhsB in killing *B. subtilis*, as evidenced by the attenuated but not abolished killing activities of the ∆*rhsB*. Further research is required to examine this penetration process in detail to elucidate the cellular death pathways using combinatorial effector mutations.

In addition, our results reveal that the AC T6SS is capable of killing a variety of target species that differ greatly in cell envelope composition and defense mechanisms. These functions are likely dependent on the T6SS‐dependent effectors, including many belonging to the Rhs family. The findings on the self‐cleavage of RhsB are consistent with previous reports that Rhs‐family effectors in *A. dhakensis* (TseI), *P. syringae* (PSPTO_5438), *P. aeruginosa* (PA2684), *V. parahaemolyticus* (VP1517), and *P. laumondii* (Rhs1) are subject to self‐cleavage^36^, ^48^. Although critical cleavage residues have been determined, the molecular mechanism remains elusive and warrants future research. In addition, it is likely that different effectors are involved in targeting different species. The effector mutant library constructed in this study will help elucidate effector specificity against different bacterial and fungal competitors in future research.

Lastly, the demonstration that the AC T6SS can kill both gram‐negative and gram‐positive bacterial and fungal species highlights the potential of developing T6SS‐based treatment strategies as green alternatives to chemical agents in mitigating infectious diseases in agricultural and medical applications. In short, there seems to be no barrier too thick for the T6SS to break in the microbial world.

## MATERIALS AND METHODS

### Bacterial strains and plasmids

Plasmids and strains are listed in Table S3. All strains were routinely grown in LB, 7H9, 7H10, or YPD media following standard culturing conditions for each species. Antibiotics were used as follows: kanamycin (25 µg/ml for bacterial strains, and 100 µg/ml for fungal strains), chloramphenicol (10 µg/ml), irgasan (25 µg/ml), gentamicin (20 µg/ml), and ampicillin (50 µg/ml). Gene expression vectors were constructed as previously described^54^. Full‐length RhsB, RhsB^CT^, or its mutants were cloned into the pBAD‐expression vector, respectively. Cells harboring different constructs were grown in LB supplemented with 0.2% (w/v) glucose to repress gene expression. All constructs were confirmed by sequencing. All primers and plasmids are available upon request.

### Bacterial competition assay

For AC intraspecies competition, exponential‐phase killer cells and stationary‐phase prey cells were mixed at a ratio of 20:1 (killer:prey) and spotted on LB agar plates for 24 h at 28°C. For the interspecies competition with *M. smegmatis*, exponential‐phase killer cells and stationary‐phase prey cells were mixed at a ratio of 2:1 (killer:prey), spotted on 7H10 plates, and co‐incubated for 8 h at 37°C. For the interspecies competition with other strains, exponential‐phase killer cells and stationary‐phase prey cells were mixed at a ratio of 10:1 (killer:prey), spotted on LB agar plates, and co‐incubated for 10 h at 28°C (for *S. cerevisiae*), or 3 h at 28°C (for *P. syringae* and all the fungal prey cells), or 3 h at 37°C (for all other bacterial prey cells) unless stated otherwise. For the competition assay in liquid media, killer and prey cells were mixed with the same ratio used in the competition assay on solid media and incubated in liquid LB for 3 h at 37°C with shaking. Survival of prey cells was quantified by serial dilution and plating on selective media. For the relative survival in Figure 3D, it was calculated by using the final survival of the specific prey cells attacked by wild type divided by the survival of the same prey cells competed with the ∆*tssM* mutant. For the relative survival in Figure 4B,G, it was calculated by using the final survival of the prey cells competed with different T6SS‐active killer strains (T6SS^+^) divided by the survival of prey cells competed with the ∆*tssM* mutant. Error bars show standard deviation of at least three biological replicates.

### Competition assay *in planta*

For competition assay using *N. benthamiana*, 4–5 weeks old plants were inoculated on the second and third leaves. For competition assay using *C. lanatus* “Jingxin No. 2”, 2‐week‐old plants were inoculated on the cotyledons. Selected regions were injected with bacterial mixtures, AC (OD_600_ ~ 5) mixed with prey cells (OD_600_ ~ 0.5) as indicated, into the abaxial side of the leaves. After 6 h infection at room temperature, discs  of infiltrated areas were collected and quickly frozen by liquid nitrogen, followed by grinding. The samples were then resuspended in liquid LB. Prey cells were quantified by ten‐fold serial dilution and plating on selective media. Error bars show standard deviation of at least three biological replicates.

### Western blot analysis

Proteins were run on a SDS‐PAGE (sodium dodecyl sulfate–polyacrylamide gel electrophoresis) gel and transferred to a PVDF (polyvinylidene fluoride) membrane (Bio‐Rad) by electrophoresis. The membrane was blocked with 5% (w/v) nonfat milk in TBST buffer (50 mM Tris, 150 mM NaCl, 0.1% (v/v) Tween‐20, pH 7.6) at room temperature for 1 h, followed by sequential treatment with primary antibodies and secondary HRP‐conjugated antibodies. Signals were detected using the Clarity Western ECL substrate (Bio‐Rad). Monoclonal antibodies to epitope tags were ordered from ABclonal (Product # AE005 [FLAG] and # AE003 [6His]), Thermo Scientific (Product # 37‐7500 [V5]), and Biolegend (Product # 663905 [RpoB]). The polyclonal antibody to Hcp (Aave_1465) was customized by Shanghai Youlong Biotech. Secondary antibodies were ordered from ZSGB‐Bio (Product # ZB‐2305 [mouse] and # ZB‐2301 [rabbit]).

### Protein secretion assay

Cultures were grown aerobically in liquid LB at 28°C to OD_600_ ~2 and harvested by centrifugation at 2500*g* for 8 min. Pellets were resuspended in fresh LB and incubated at 28°C for 1 h without shaking. Cells were spinned down twice at room temperature at 10,000*g* for 2 min. Pellets were resuspended in SDS‐loading buffer (Epizyme Biotech) and used as whole‐cell samples. Supernatants were precipitated in TCA (trichloroacetic acid, 20% [v/v]) at −20°C for 30 min and centrifuged at 15,000*g* for 30 min at 4°C. The pellets were then washed with acetone and the air‐dried pellets were resuspended in SDS‐loading buffer. Whole‐cell and secretion samples were boiled for 10 min prior to SDS‐PAGE and Western blot analysis. For secretome analysis, the supernatants of overnight cultures were collected and subject to SDS‐PAGE analysis. Gel slices containing secreted proteins were cut out and sent for LC‐MS/MS analysis performed by the Instrumental Analysis Center of Shanghai Jiao Tong University.

### Protein purification and enzymatic assay

RhsB and its variants were expressed using the pETSUMO vectors in *E. coli* BL21(DE3). Cells were grown in liquid LB medium to exponential phase (OD_600_ ~ 0.6) at 37°C. For the expression of His‐SUMO‐RhsB^CT^ or its non‐toxic mutants, cells were induced with 1 mM IPTG at 37°C for 5 h. For the expression of His‐SUMO‐RhsB^KE‐AA^, cells were induced with 1 mM IPTG at 20°C for 18 h. The cells were harvested by centrifugation at 4500*g* for 10 min. The pellets were resuspended in lysis buffer (20 mM Tris‐HCl, 150 mM NaCl, 10 mM imidazole, pH 8.0) and lysed by sonication. Lysates were centrifuged at 15,000*g* for 20 min and the supernatants were incubated with Ni‐NTA resin (Smart‐lifesciences). Proteins were eluted in elution buffer (20 mM Tris‐HCl, 150 mM NaCl, and variable concentrations of imidazole, pH 8.0). Eluted samples were analyzed by SDS‐PAGE analysis.

Protein activity *in vitro* was detected by incubating with 100 ng plasmid at 37°C for 1 h. NEB CutSmart buffer (50 mM potassium acetate, 20 mM tris‐acetate, 10 mM magnesium acetate, 100 µg/ml bovine serum albumin, pH 7.9) was chosen as the reaction buffer. Purified proteins (0.1 μg) and 0.5 units DNase I (positive control) were used separately in each reaction.

### Protein pull‐down assay

Genes of interest were cloned into pET and pBBRT vectors for expression. Cells were grown in liquid LB medium with appropriate antibiotics to exponential phase (OD_600_ ~ 0.6) at 37°C and induced with 1 mM IPTG overnight at 20°C for pET vectors or 100 ng/ml anhydrotetracycline (aTc) for 3 h at 37°C for pBBRT vectors. Cells were harvested by centrifugation at 4500*g* for 10 min, resuspended in lysis buffer (20 mM Tris, pH 8.0, 500 mM NaCl, 50 mM imidazole with protease inhibitor [Thermo Scientific]), and lysed by sonication. After cell debris was removed by centrifugation at 15,000*g* for 5 min, supernatants were mixed and incubated with Ni‐NTA resin (Smart‐lifesciences) at 4°C for 1 h. The samples were then washed five times with wash buffer (20 mM Tris, pH 8.0, 500 mM NaCl, 50 mM imidazole), and eluted in elution buffer (20 mM Tris, pH 8.0, 500 mM NaCl, 500 mM imidazole). Input and eluted samples were boiled for 10 min prior to SDS‐PAGE and Western blot analysis.

### Protein toxicity assay

Cells harboring different plasmids were grown on LB agar plates with appropriate antibiotics and 0.2% (w/v) glucose at 30°C overnight. Cells were then harvested and resuspended in fresh liquid LB medium and grown to OD_600_ = 1. A series of ten‐fold dilutions were plated on LB agar plates containing 0.1% (w/v) L‐arabinose or 0.2% (w/v) glucose for induction and repression, respectively. For Figure 4I, the cells were induced with 0.1% (w/v) L‐arabinose for 2 h. The survival of *E. coli* before and after induction was enumerated by ten‐fold dilutions on LB agar plates containing 0.2% (w/v) glucose and appropriate antibiotics. Each experiment was performed at least two times, with one representative experiment shown.

### Bacterial two‐hybrid assay

Proteins were fused to the T25 and the T18 domains of the *Bordetella* adenylate cyclase as previously described^54^. Plasmids encoding fusion proteins were co‐expressed in the reporter strain BTH101. Single colonies for each transformation were inoculated into 300 μl of LB medium, respectively, and grown for 4 h at 30°C with shaking. Each culture (3 μl) was spotted onto LB agar plates supplemented with kanamycin, ampicillin, IPTG (0.02 mM), and X‐Gal (40 µg/ml). Plates were incubated for 6 h at 30°C and then 10 h at room temperature. The experiments were performed in triplicate and a representative result is shown.

### Bioinformatic and statistical analysis

Gene sequences of *A. citrulli* AAC00‐1 were downloaded from GenBank NC_008752.1. DNA sequences were managed and analyzed by Benchling. RhsB sequence was analyzed with Phyre2^55^. The model figure was generated using BioRender (https://biorender.com). Statistical analysis was performed using the GraphPad Prism software (9.3.0). The statistical significance was evaluated using a two‐tailed Student's *t*‐test or one‐way ANOVA test as indicated.

### N‐terminal Edman sequencing

Purified proteins were subjected to an SDS‐PAGE gel. Protein bands corresponding to RhsB M‐fragment and C‐terminus were excised individually and sent for Edman sequencing that was performed at the BiotechPack Scientific Company.

## AUTHOR CONTRIBUTIONS

Tao Dong conceived the project. Tong‐Tong Pei, Yumin Kan, Zeng‐Hang Wang, Shuangquan Yan, Ming‐Xuan Tang, Hao Li, Yang Cui, Han Luo, Hao‐Yu Zheng, and Xiaoye Liang performed research; Tao Dong and Tong‐Tong Pei wrote the manuscript.

## ETHICS STATEMENT

There is no animal used in this study.

## CONFLICT OF INTERESTS

The authors declare that they have no competing interests.

## Supporting information

Supporting information.

Supporting information.

Supporting information.

Supporting information.

Supporting information.

## Data Availability

Data supporting the findings of this study are available within the paper or from the corresponding author upon request. Request for materials should be addressed to the corresponding author.
