# Supplementary material for: Delivery of an Rhs‐family nuclease effector reveals direct penetration of the gram‐positive cell envelope by a type VI secretion system in Acidovorax citrulli
Source: mLife. 2022 Mar 24;1(1):66–78. doi: 10.1002/mlf2.12007 (PMC10989746; doi:10.1002/mlf2.12007)
Supplement: Supplementary file 1 — Supporting information. [file MLF2-1-66-s005.pdf]

Supplementary Information

Delivery of an Rhs-family nuclease effector reveals direct penetration of the gram-positive cell envelope by a type VI secretion system in *Acidovorax citrulli*

Pei et al.

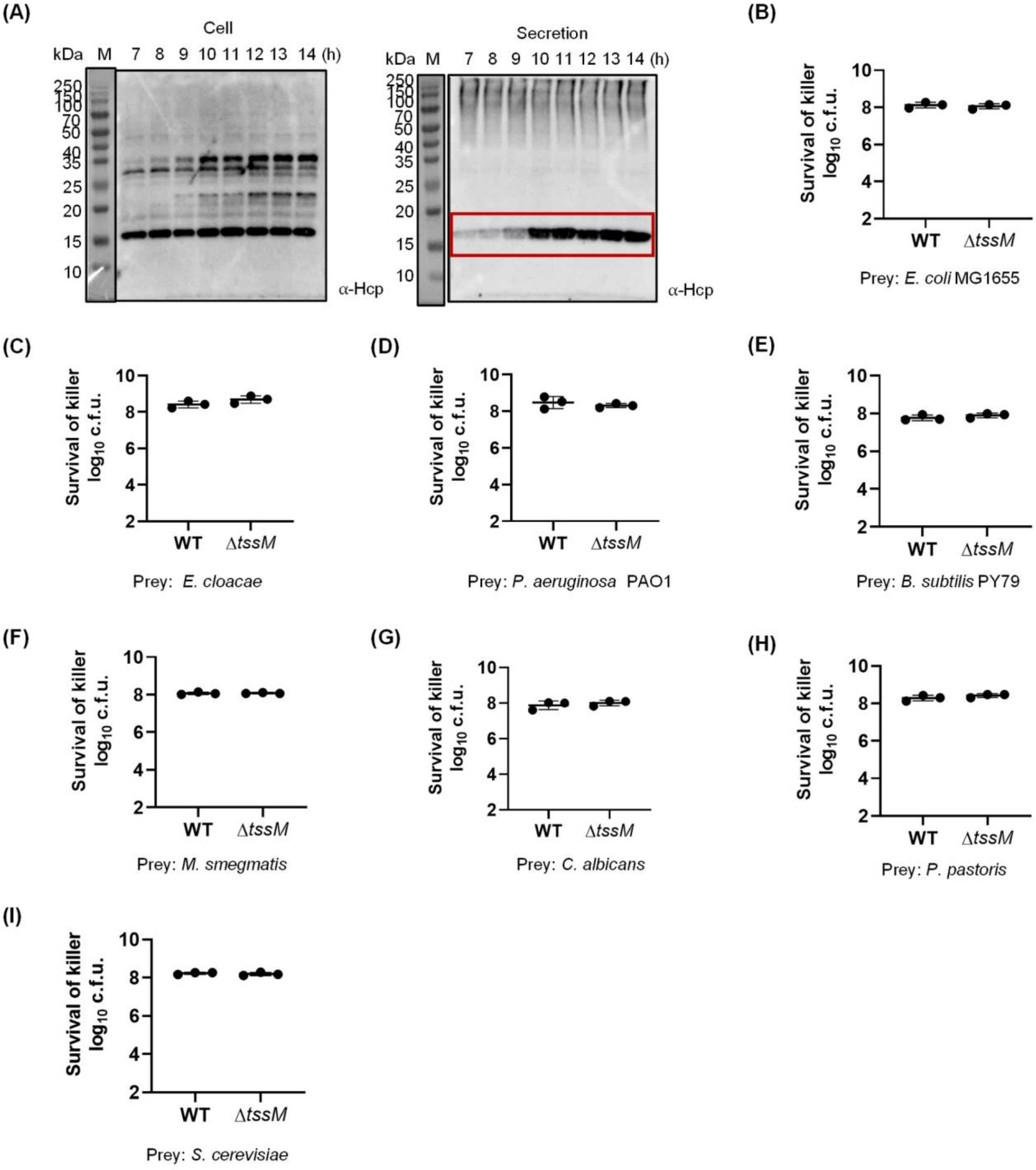

Supplementary Fig. 1. Activities of *A. citrulli* T6SS in secretion and bacterial competition. (A) Full images of Hcp secretion of AC in Figure 1A. (B) to (I) Survival of killer strains during competition assays for which the survival of the prey is shown in Figure 1B and 1C, respectively. Error bars indicate the standard deviation of three biological replicates.

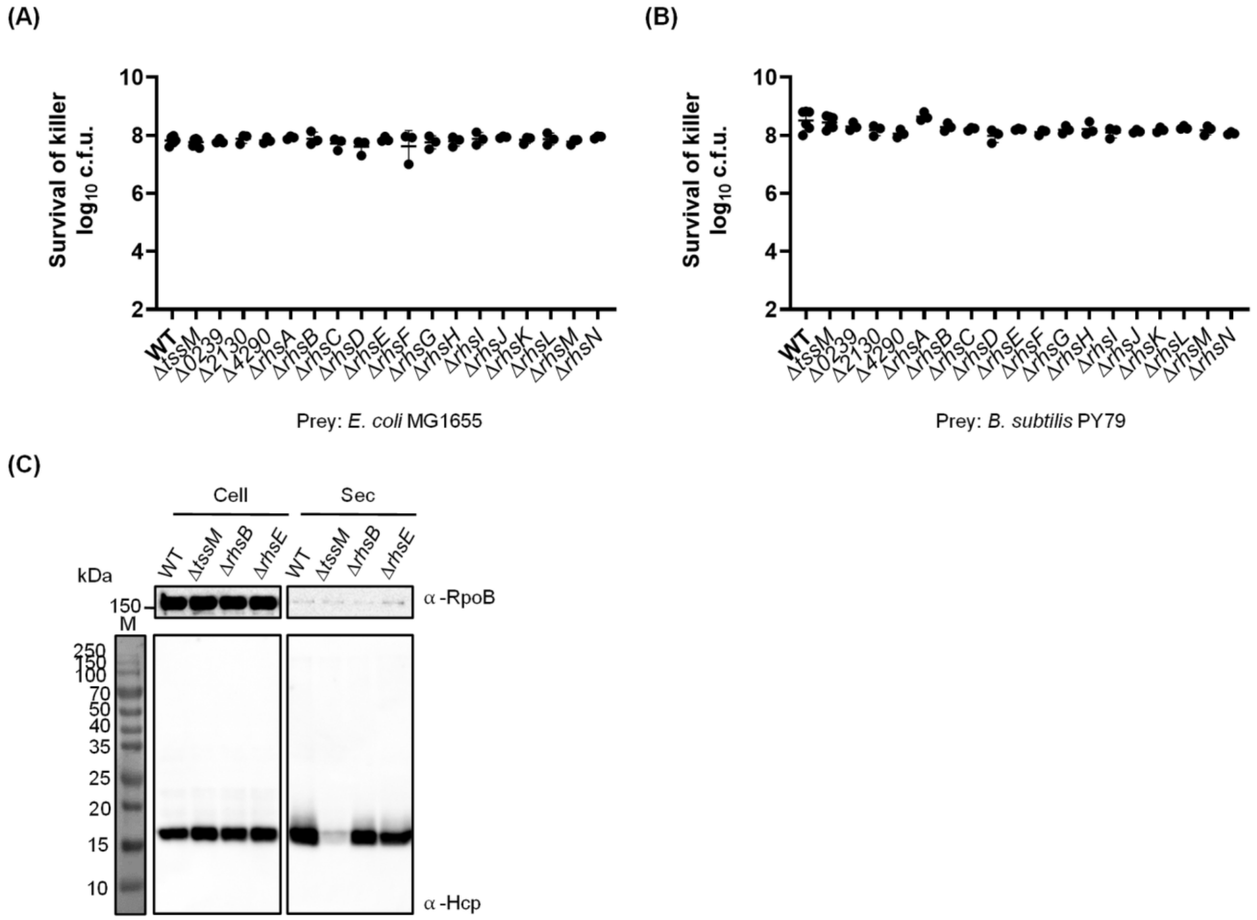

Supplementary Fig. 2. Effects of *A. citrulli* effector deletion or inactivating insertion. (A) and (B) Survival of killer strains during competition assays for which the survival of the prey is shown in Figure 2D and 2E, respectively. Error bars indicate the standard deviation of at least three biological replicates. (C) Secretion analysis of Hcp in the WT, Δ*tssM*, Δ*rhsB*, and Δ*rhsE*. RpoB serves as an equal loading and autolysis control.

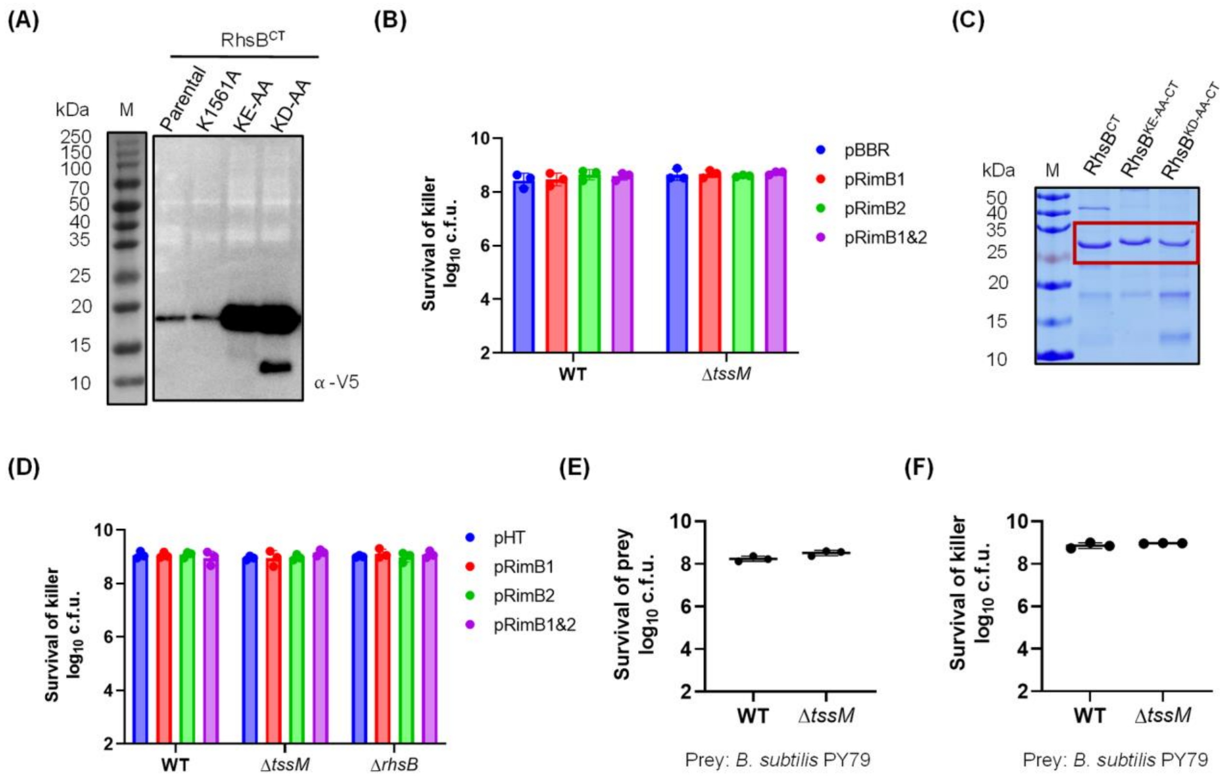

Supplementary Fig. 3. Characterization of RhsB activity. (A) Western blot analysis confirming plasmid-borne RhsB^{CT} and its catalytic mutants tested in Figure 3B were expressed. Wild-type RhsB^{CT} and K1561A mutant were barely detected, likely due to low abundance resulting from toxicity. (B) Survival of killer strains during competition assays for which the survival of the prey is shown in Figure 3D. (C) SDS-PAGE analysis of purified His-SUMO-RhsB^{CT} and its catalytic mutant proteins used in Figure 3F. (D) Survival of killer strains during competition assays for which the survival of the prey is shown in Figure 3G. (E) Competition assay performed in liquid media of WT and Δ*tssM* against *B. subtilis* PY79. Survival of killer strains during competition assays is shown in (F). Error bars indicate the standard deviation of three biological replicates.

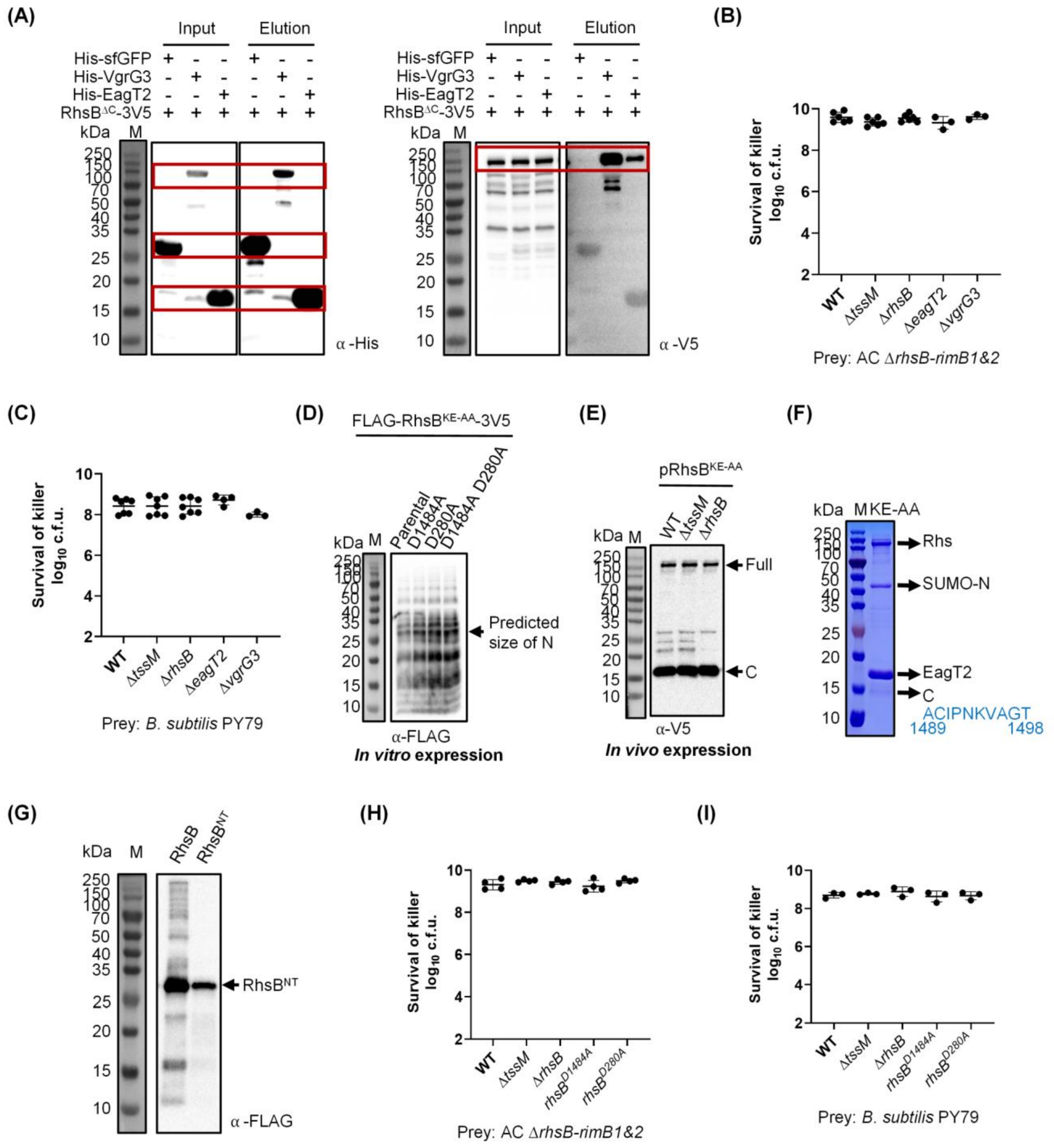

Supplementary Fig. 4. Delivery and cleavage of RhsB. (A) Full images of the pull-down analysis in Figure 4A. (B) and (C) Survival of killer strains during competition assays for which the survival of the prey is shown in Figure 4B and 4C. (D) Western blot analysis showing the N-terminal signals of *in vitro* expression of RhsB$^{KE\text{-}AA}$ and its cleavage-defective mutants D1484A, D280A, and D1484A D280A. The

signals of C-terminus are shown in Figure 4F. (E) Western blot analysis showing the signals of C-terminal 3V5-tagged RhsB$^{KE\text{-}AA}$ expressed in AC WT, T6SS-null mutant Δ*tssM*, and Δ*rhsB*, respectively. (F) SDS-PAGE analysis of purified His-SUMO-tagged RhsB$^{KE\text{-}AA}$ with His-tagged EagT2. C-terminal cleavage site was determined by N-terminal sequencing and the identified peptide sequence is indicated. (G) Western blot analysis showing the signals of N-terminal FLAG-tagged full-length RhsB and predicted N-terminal RhsB (RhsB^{NT}). (H) and (I) Survival of killer strains during competition assays for which the survival of the prey is shown in Figure 4G and 4H. Error bars indicate the standard deviation of at least three biological replicates.

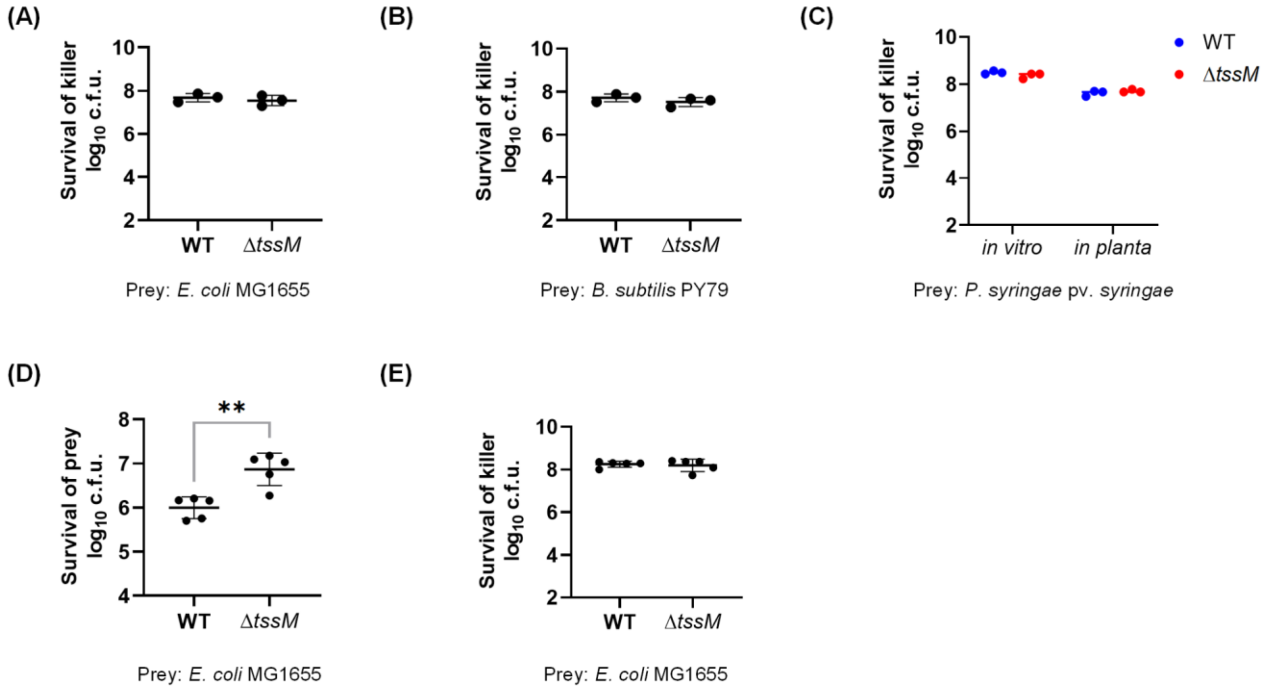

Supplementary Fig. 5. Survival of *A. citrulli* strains during *in planta* bacterial competition. (A) to (C) Survival of killer strains during competition assays for which the survival of the prey is shown in Figure 5A to 5C, respectively. Survival of prey (D) and killer (E) for the competition assay of WT and Δ*tssM* against *E. coli* MG1655 *in planta* using *C. lanatus*. Error bars indicate the standard deviation of at least three biological replicates and statistical significance was calculated using a two-tailed Student's *t*-test for each group, $^{**}p < 0.01$.
