# Supplementary material for: Delivery of an Rhs‐family nuclease effector reveals direct penetration of the gram‐positive cell envelope by a type VI secretion system in Acidovorax citrulli
Source: mLife. 2022 Mar 24;1(1):66–78. doi: 10.1002/mlf2.12007 (PMC10989746; doi:10.1002/mlf2.12007)
Supplement: Supplementary file 5 — Supporting information. [file MLF2-1-66-s004.pdf]

Table S3. Plasmids and strains.

Plasmid	Description	Reference
pEXG2.0	Suicidal conjugation vector for all chromosomal allelic changes	Lab stock
pEXG2.0-Ac0239+imm	Suicidal vector to construct in-frame deletion mutant of Aave_0239 and the predicted immunity gene	This study
pEXG2.0-Ac0483	Suicidal vector to construct inactivating-insertion in Aave_0483	This study
pEXG2.0-Ac0499	Suicidal vector to construct inactivating-insertion in Aave_0499	This study
pEXG2.0-Ac2049+imm	Suicidal vector to construct in-frame deletion mutant of Aave_2049 and the predicted immunity gene	This study
pEXG2.0-Ac2130	Suicidal vector to construct inactivating-insertion in Aave_2130	This study
pEXG2.0-Ac2740+imm	Suicidal vector to construct in-frame deletion mutant of Aave_2740 and the predicted immunity gene	This study
pEXG2.0-Ac2838+imm	Suicidal vector to construct in-frame deletion mutant of Aave_2838 and the predicted immunity gene	This study
pEXG2.0-Ac3345+imm	Suicidal vector to construct in-frame deletion mutant of Aave_3345 and the predicted immunity gene	This study
pEXG2.0-Ac3478	Suicidal vector to construct inactivating-insertion in Aave_3478	This study
pEXG2.0-Ac3481+imm	Suicidal vector to construct in-frame deletion mutant of Aave_3481 and the predicted immunity gene	This study
pEXG2.0-Ac3751+imm	Suicidal vector to construct in-frame deletion mutant of Aave_3751 and the predicted immunity gene	This study
pEXG2.0-Ac3788	Suicidal vector to construct inactivating-insertion in Aave_3788	This study
pEXG2.0-Ac3794	Suicidal vector to construct inactivating-insertion in Aave_3794	This study
pEXG2.0-Ac3796	Suicidal vector to construct inactivating-insertion in Aave_3796	This study
pEXG2.0-Ac4008	Suicidal vector to construct inactivating-insertion in Aave_4008	This study
pEXG2.0-Ac4283	Suicidal vector to construct inactivating-insertion in Aave_4283	This study
pEXG2.0-Ac4290	Suicidal vector to construct in-frame deletion mutant of Aave_4290	This study
pEXG2.0-Ac-d0499+2imm	Suicidal vector to construct in-frame deletion mutant of Aave_0499 and the predicted immunity genes	This study
pEXG2.0-Aave0498	Suicidal vector to construct in-frame deletion mutant of Aave_0498	This study
pEXG2.0-Aave0497	Suicidal vector to construct in-frame deletion mutant of Aave_0497	This study
pEXG2.0-Aave0499$^{KE\text{-}AA}$	Suicidal vector to construct Aave_0499 $^{KE\text{-}AA}$ mutant	This study
pMV261-hsp60-LacZ	Temperature Inducible *E. coli* -*Mycobacterium* shuttle plasmid of pMV261 with lacZ fusion, kanamycin resistance	Lab stock
pBAD18kan	Arabinose inducible expression vector, kanamycin resistance	[1]
pBAD24kan-0499^{C}-3V5	Arabinose inducible expression of Aave_0499 with a C-terminal 3V5 tag	This study
pBAD24kan-0499$^{C\ K1561A}$-3V5	Arabinose inducible expression of Aave_0499 $^{C\ K1561A}$ with a C-terminal 3V5 tag	This study

pBAD24kan-0499$^{\text{C KE-AA}}$-3V5	Arabinose inducible expression of Aave_0499$^{\text{C KE-AA}}$ with a C-terminal 3V5 tag	This study
pBAD24kan-0499$^{\text{C KD-AA}}$-3V5	Arabinose inducible expression of Aave_0499$^{\text{C KD-AA}}$ with a C-terminal 3V5 tag	This study
pBAD24kan-0499	Arabinose inducible expression of Aave_0499	This study
pBAD24kan-0499-3V5	Arabinose inducible expression of Aave_0499 with a C-terminal 3V5 tag	This study
pBAD24kan-0499$^{\text{D280A}}$-3V5	Arabinose inducible expression of Aave_0499$^{\text{D280A}}$ with a C-terminal 3V5 tag	This study
pBAD24kan-0499$^{\text{D1484A}}$-3V5	Arabinose inducible expression of Aave_0499$^{\text{D1484A}}$ with a C-terminal 3V5 tag	This study
pBAD24kan-0499$^{\text{D1484A D280A}}$-3V5	Arabinose inducible expression of Aave_0499$^{\text{D1484A D280A}}$ with a C-terminal 3V5 tag	This study
pBAD24kan-FLAG-RhsB-3V5	Arabinose inducible expression of Aave_0499 with an N-terminal FLAG tag and a C-terminal 3V5 tag	This study
pBAD24kan-FLAG-RhsB^{NT}	Arabinose inducible expression of RhsB^{NT} with an N-terminal FLAG tag	This study
pBAD33	Arabinose inducible expression vector, chloramphenicol resistance	Lab stock
pBAD33-RimB1	Arabinose inducible expression of RimB1	This study
pBAD33-RimB2	Arabinose inducible expression of RimB2	This study
pBAD33-RimB1-RimB2	Arabinose inducible expression of RimB1 and RimB2	This study
pBBR1MCS-5	A broad-host-range cloning vector, gentamicin resistance	Lab stock
pBBR1MCS-2	A broad-host-range cloning vector, kanamycin resistance	Lab stock
pBBR1MCS-2-RimB1	Constitutive expression of RimB1	This study
pBBR1MCS-2-RimB1	Constitutive expression of RimB2	This study
pBBR1MCS-2-RimB1-RimB2	Constitutive expression of RimB1 and RimB2	This study
pBBR1MCS-2-RimB$^{\text{KE-AA}}$-3V5	Constitutive expression of RimB$^{\text{KE-AA}}$ with a C-terminal 3V5 tag	
pKNT25-RhsB$^{\text{KE-AA}}$	IPTG inducible expression of RhsB$^{\text{KE-AA}}$ for bacterial two-hybrid analysis	This study
pKNT25-RimB1	IPTG inducible expression of RimB1 for bacterial two-hybrid analysis	This study
pKNT25-RimB2	IPTG inducible expression of RimB2 for bacterial two-hybrid analysis	This study
pKNT25-0500	IPTG inducible expression of Aave_0500 for bacterial two-hybrid analysis	This study
pKNT25-Pal	IPTG inducible expression of Pal for bacterial two-hybrid analysis	This study
pCH363-RhsB$^{\text{KE-AA}}$	IPTG inducible expression of RhsB$^{\text{KE-AA}}$ for bacterial two-hybrid analysis	This study
pCH363-RimB1	IPTG inducible expression of RimB1 for bacterial two-hybrid analysis	This study
pCH363-RimB2	IPTG inducible expression of RimB2 for bacterial two-hybrid analysis	This study
pCH363-0500	IPTG inducible expression of Aave_0500 for bacterial two-hybrid analysis	This study
pCH363-TolB	IPTG inducible expression of TolB for bacterial two-hybrid analysis	This study
p15a	anhydrotetracycline(aTc) inducible expression vector, chloramphenicol resistance	Lab stock

pBBRT	aTc inducible plasmid by inserting the tetO induction system from p15a, kanamycin resistance	This study
pBBRT -RhsB$^{\Delta C}$-3V5	aTc inducible expression of Aave_0499$^{\Delta C}$ with a C-terminal 3V5 tag	This study
pETSUMO-0499^{CT}	IPTG inducible expression of 0499^{CT} with an N-terminal His-SUMO tag	This study
pETSUMO-0499$^{KE\text{-}AA\text{-}CT}$	IPTG inducible expression of Aave_0499$^{KE\text{-}AA\text{-}CT}$ with an N-terminal His-SUMO tag	This study
pETSUMO-0499$^{KD\text{-}AA\text{-}CT}$	IPTG inducible expression of Aave_0499$^{KD\text{-}AA\text{-}CT}$ with an N-terminal His-SUMO tag	This study
pETDUET-His-VgrG3	IPTG inducible expression of VgrG3 with an N-terminal His tag	This study
pETDUET-His-EagT2	IPTG inducible expression of EagT2 with an N-terminal His tag	This study
pET22b-FLAG-RhsB$^{KE\text{-}AA}$-3V5	IPTG inducible expression of RhsB$^{KE\text{-}AA}$ with an N-terminal FLAG tag and a C-terminal 3V5 tag	This study
pET22b-FLAG-RhsB$^{D280A\ KE\text{-}AA}$-3V5	IPTG inducible expression of RhsB $^{D280A\ KE\text{-}AA}$ with an N-terminal FLAG tag and a C-terminal 3V5 tag	This study
pET22b-FLAG-RhsB$^{D1484A\ KE\text{-}AA}$-3V5	IPTG inducible expression of RhsB $^{D1484A\ KE\text{-}AA}$ with an N-terminal FLAG tag and a C-terminal 3V5 tag	This study
pET22b-FLAG-RhsB$^{D280A\ D1484A\ KE\text{-}AA}$-3V5	IPTG inducible expression of RhsB $^{D280A\ D1484A\ KE\text{-}AA}$ with an N-terminal FLAG tag and a C-terminal 3V5 tag	This study
pET28a-sfGFP	IPTG inducible expression of sfGFP with an N-terminal His tag	Lab stock
pHT01	IPTG Inducible *E. coli* - *B. subtilis* shuttle plasmid, chloramphenicol resistance in *B. subtilis*	Lab stock
pHT01-RimB1	IPTG inducible expression of RimB1	This study
pHT01-RimB2	IPTG inducible expression of RimB2	This study
pHT01-RimB1-RimB2	IPTG inducible expression of RimB1 and RimB2	This study

**Strain**	**Genotype**	**Description**	**Reference**
Acidovorax citrulli AAC00-1	Parental	Parental, reference strain of Group II strain	Lab stock
	Δ*tssM*	T6SS null, in-frame deletion of *tssM*	This study
	Δ*0239*	In-frame deletion of Aave_0239 and the predicted immunity gene	This study
	Δ*rhsA*	Inactivating-insertion in Aave_0483 with pEXG2.0	This study
	Δ*rhsB*	Inactivating-insertion in Aave_0499 with pEXG2.0	This study
	Δ*rhsC*	In-frame deletion of Aave_2049 and the predicted immunity gene	This study
	Δ*2130*	Inactivating-insertion in Aave_2130 with pEXG2.0	This study
	Δ*rhsD*	In-frame deletion of Aave_2740 and the predicted immunity gene	This study
	Δ*rhsE*	In-frame deletion of Aave_2838 and the predicted immunity gene	This study
	Δ*rhsF*	In-frame deletion of Aave_3345 and the predicted immunity gene	This study
	Δ*rhsH*	Inactivating-insertion in Aave_3478 with pEXG2.0	This study
	Δ*rhsG*	In-frame deletion of Aave_3481 and the predicted immunity gene	This study
	Δ*rhsI*	In-frame deletion of Aave_3751 and the predicted immunity gene	This study
	Δ*rhsJ*	Inactivating-insertion in Aave_3788 with pEXG2.0	This study
	Δ*rhsK*	Inactivating-insertion in Aave_3794 with pEXG2.0	This study
	Δ*rhsL*	Inactivating-insertion in Aave_3796 with pEXG2.0	This study
	Δ*rhsM*	Inactivating-insertion in Aave_4008 with pEXG2.0	This study
	Δ*rhsN*	Inactivating-insertion in Aave_4283 with pEXG2.0	This study
	Δ*4290*	In-frame deletion of Aave_4290	This study
	Δ*rhsB-rimB1&2*	In-frame deletion of Aave_0499 and the predicted immunity genes	This study
	$rhsB^{KE\text{-}AA}$	Chromosomal mutation of the RhsB catalytic residues K1571 and E1572	This study
	$rhsB^{D280A}$	Chromosomal mutation of the RhsB catalytic residues D280	This study
	$rhsB^{D1484A}$	Chromosomal mutation of the RhsB catalytic residues D1484	This study
	Δ*vgrG3*	In-frame deletion of Aave_0497	This study
	Δ*eagT2*	In-frame deletion of Aave_0498	This study
E. coli			
T-Fast		Strain used for cloning and gene expression	TIANGEN

WM6026		Strain used for conjugation	Lab stock
BTH101		Host strain used for two-hybrid analysis	[2]
BL21(DE3)		Strain used for protein expression	Lab stock
MG1655		Strain used for competition assay	Lab stock
M. smegmatis mc^{2}155		Strain used for competition assay	Lab stock
Bacillus subtilis PY79		Strain used for competition assay	Lab stock
Pseudomonas aeruginosa PAO1		Strain used for competition assay	Lab stock
Enterobacter cloacae B29		Strain used for competition assay	Lab stock
Candida albicans		Strain used for competition assay	Lab stock
Pichia pastoris X33		Strain used for competition assay	Lab stock
Saccharomyces cerevisiae BY4741		Strain used for competition assay	Lab stock

Note for references

1. Guzman, L. M., Belin, D., Carson, M. J. & Beckwith, J. Tight regulation, modulation, and high-level expression by vectors containing the arabinose PBAD promoter. *J. Bacteriol.* **177**, 4121–4130 (1995).
2. Battesti, A. & Bouveret, E. The bacterial two-hybrid system based on adenylate cyclase reconstitution in *Escherichia coli*. *Methods* **58**, 325–334 (2012).
